# Editorial: Advanced neural stem cell therapies for spinal cord injury

**DOI:** 10.3389/fphar.2024.1469535

**Published:** 2024-08-06

**Authors:** Venkatesh Katari, Santhosh Kumar Pasupuleti, Madhubanti Mullick, Vinod Kumar Reddy Lekkala, Dwaipayan Sen

**Affiliations:** ^1^ Department of Physiology and Pharmacology, College of Medicine and Life Sciences, The University of Toledo, Toledo, OH, United States; ^2^ Department of Pediatrics, Herman B Wells Center for Pediatric Research, Indiana University School of Medicine, Indianapolis, IN, United States; ^3^ Department of Cellular and Molecular Medicine, University of California, San Diego, San Diego, CA, United States; ^4^ Department of Ophthalmology, University of California, San Francisco, San Francisco, CA, United States; ^5^ Cell And Gene Therapies, Innovative Medicines Accelerator (IMA), Stanford University, Palo Alto, CA, United States

**Keywords:** neural stem cells, 3D scaffolds, Mesenchymal stem cells, neural regeneration, spinal cord injury

This edition of Frontiers in Pharmacology is dedicated to sharing the progress in spinal cord injury research. A total of six articles are published in this Research Topic “*Advanced Neural Stem Cell Therapies for Spinal Cord Injury*”. Shang et al. provided a comprehensive analysis of the current research status of spinal cord injury using the stem cell therapy. The findings provide a foundation for future research and clinical translation efforts of stem cell therapy in SCI research field. Guo et al. provided a thorough bibliometric and visualized research hotspots and trend analysis of cell transplantation in traumatic SCI. Sadaf et al. demonstrated the potential applicability of nobiletin a polymethoxylated flavonoid with anti-oxidative and anti-inflammatory effects as a neuroprotectant against chemical-induced neurotoxicity. Winn et al. provided a proof-of-concept using induced-pluripotent stem cell derived neuroepithelial stem cells as potential therapeutics for SCI. Sintakova and Romanyuk and Rybachuk et al. provided a comprehensive reviews on therapeutic role of extracellular vesicles, microRNA, neural stem cells (NSCs) embedded-hydrogels towards SCI therapy respectively.

Spinal cord injury (SCI) is a devastating disease resulting from traumatic or non-traumatic conditions. SCI results in a range of complications, depending on the severity and location of injury. The potential consequences include physical impairment, paralysis, bowel, bladder and sexual dysfunction (Venkatesh, Ghosh et al., 2019). The annual prevalence of traumatic SCI is around 54 cases per one million people, and 18,000 new SCI cases each year are being recorded in the United States. The estimated number of people with SCI is approximately ranging from 255,000 to 383,000 in United states, (National Spinal Cord Injury Statistical Center, University of Alabama at Birmingham, 2024). The average of affected person’s age has increased from 29 years during the 1970s to 43 since 2015 About 78% of new SCI cases since 2015 belong to male category (Ding, Hu et al., 2022). Vehicle accidents are the most recent leading cause of SCI injury, followed by falls, acts of violence (primarily gunshot wounds) and sports/recreation activities are also relatively common causes. Therefore, SCI patients face significant financial and health burdens. The estimated lifetime economic burden per individual with SCI from 1.5 million to 3.0 million. These burdens highlight the importance of effective prevention, treatment, rehabilitation, and ongoing healthcare to alleviate the global burden of SCI.

## Stem cell therapy approach

Stem cell therapy is a promising area of research for the treatment of SCI. Stem cell transplantation involves injecting stem cells into the damaged area to promote healing. Studies have shown that stem cell therapy is safe and may benefit people with spinal cord injuries. Several clinics have launched phase 1/2 randomized clinical trials of stem cells for treating patients with severe SCI. Among stem cells, Mesenchymal Stem Cells (MSCs) appear to be the most therapeutic for SCI or other neurological disorders (Venkatesh and Sen, 2017). While there is hope, there is currently no firm evidence that stem cell therapy can cure SCI. Another stem cell source is NSCs, which are multipotent stem cells that can regenerate three types of cells such as neurons, astrocytes, and oligodendrocytes. This unique feature makes NSCs appropriate therapeutic candidates for treating injured spinal cord or various neurological diseases.

## Neural stem cells

The major problem of NSCs using in clinics is their availability. Alternatively, NSCs are shown to produce in large quantities using MSCs, iPSCs, embryonic stem cells (ESCs) (Venkatesh, Reddy et al., 2017; Venkatesh, Kumari et al., 2019). NSCs have significant implications in the treatment of SCI. The advantages of using NSCs over other stem cells are, they promote a. Regeneration and repair: NSCs can regenerate three types of neural and glial cells and enable migration at the damaged site and form myelin sheath and the regeneration of demyelinated axons. This makes NSCs a promising cell source for rebuilding tripartite junctions and initiating neural signals. b. Enhanced locomotor functions: NSCs are reported to enhance the recovery of locomotor function in mice after SCI, by means of replacing the damaged neurons and secreting neurotrophic molecules. c. Recruitment of endogenous NSCs: After SCI, the endogenous NSCs of heterogeneous nature are activated and proliferate and migrate towards the lesion site and mainly differentiate into astrocytes to repair the injured tissue. It is important to note that while NSCs show promise in the treatment of SCI, more research is needed to fully understand their potential and to develop safe and effective therapeutic strategies.

## Neural stem cell transplantation challenges

Transplantation of NSCs for SCI treatment holds great promise, but it also presents several challenges: i. survival and integration: Ensuring the NSC survival and integration at the damaged site is a major challenge due to harsh microenvironment. The harsh environment of the injured spinal cord can lead to poor survival rates of the transplanted NSCs. ii. Tri-Differentiation capability: Controlling the tri-differentiation of NSCs into neural and glial cell types is another challenge. The low differentiation efficiency of the transplanted cells can hinder the therapeutic effects of NSC transplantation. iii. Limited availability of NSC sources: The availability of NSC sources is limited, which can hinder the application of NSC transplantation for the treatment of SCI. These challenges highlight the need for further research to improve the effectiveness of NSC transplantation and production of clinical-grade NSCs for the treatment of SCI.

## Advanced technologies in NSC stem cell therapy for SCI

Advanced technologies have been developed to maintain the 3D-architecture of NSCs using biomaterials which enhances NSC proliferation and differentiation after transplantation. These provide a biocompatible environment which mimics *in vivo* tissue architecture and promote cell adhesion, self-renewal, and differentiation. Functional scaffolds provide bioactive signals to regulate harsh surrounding areas, whereas specific growth factors are coated on the biomaterials, and these are released in a controlled time-dependent manner. PEDOT-HA/Cs/Gel scaffold is designed by introducing poly (3,4-ethylenedioxythiophene) doped with hyaluronic acid (PEDOT-HA) nanoparticles into a chitosan/gelatin (Cs/Gel) matrix. These scaffolds have been shown excellent biocompatibility for NSC proliferation and differentiation. Interestingly, electroactive CP polypyrrole (PPy) is another organic material which can conduct electrical impulses. Electrical stimulation of polypyrrole dodecylbenzenesulfonate (anionic) (PPy(DBS)) induced hNSCs differentiation into β-III Tubulin (Tuj1) expressing neurons (high population), glial fibrillary acidic protein (GFAP) expressing glial cells (low population) (Stewart, Kobayashi et al., 2015). Poly(3,4-ethylenedioxythiophene) polystyrene sulfonate (PEDOT:PSS) is one such commercially available high-conductivity grade material which promotes neurogenic commitment of neural crest-derived stem cells towards differentiation of MAP-2 and β—Tubulin-III expressing neuronal cells (Pisciotta, Lunghi et al., 2022). Collagen type I is added to enhance the biomimicry potential of the surfaces. These advanced scaffolds not only support cell regeneration but also deliver cytokines and other soluble factors and most importantly these are able to transmit neural signals to the adjacent host tissues and connect host neural circuits. These materials guide and support newly growing tissues, promoting neural proliferation at the injured site. However, the development of these 3D-scaffolds requires further research in validating their safety before clinical use.

## References

[B1] Ding W. Hu S. Wang P. Kang H. Peng R. Dong Y. (2022). Spinal cord injury: the global incidence, prevalence, and disability from the global burden of disease study 2019. Spine (Phila Pa 1976) 47 (21), 1532–1540. 10.1097/BRS.0000000000004417 35857624 PMC9554757

[B2] Pisciotta A. Lunghi A. Bertani G. Di Tinco R. Bertoni L. Orlandi G. (2022). PEDOT: PSS promotes neurogenic commitment of neural crest-derived stem cells. Front. Physiol. 13, 930804. 10.3389/fphys.2022.930804 36060701 PMC9428488

[B3] Stewart E. Kobayashi N. R. Higgins M. J. Quigley A. F. Jamali S. Moulton S. E. (2015). Electrical stimulation using conductive polymer polypyrrole promotes differentiation of human neural stem cells: a biocompatible platform for translational neural tissue engineering. Tissue Eng. Part C Methods 21 (4), 385–393. 10.1089/ten.TEC.2014.0338 25296166

[B4] Venkatesh K. Ghosh S. K. Mullick M. Manivasagam G. Sen D. (2019). Spinal cord injury: pathophysiology, treatment strategies, associated challenges, and future implications. Cell. Tissue Res. 377 (2), 125–151. 10.1007/s00441-019-03039-1 31065801

[B5] Venkatesh K. Kumari A. Sen D. (2019). MicroRNA signature changes during induction of neural stem cells from human mesenchymal stem cells. Nanomedicine 17, 94–105. 10.1016/j.nano.2019.01.003 30664947

[B6] Venkatesh K. Reddy L. V. K. Abbas S. Mullick M. Moghal E. T. B. Balakrishna J. P. (2017). NOTCH signaling is essential for maturation, self-renewal, and tri-differentiation of *in vitro* derived human neural stem cells. Cell. Reprogr. 19 (6), 372–383. 10.1089/cell.2017.0009 29035086

[B7] Venkatesh K. Sen D. (2017). Mesenchymal stem cells as a source of dopaminergic neurons: a potential cell based therapy for Parkinson's disease. Curr. Stem Cell. Res. Ther. 12 (4), 326–347. 10.2174/1574888X12666161114122059 27842480

